# Proteomics for heart failure risk stratification: a systematic review

**DOI:** 10.1186/s12916-024-03249-7

**Published:** 2024-01-25

**Authors:** Kayode O. Kuku, Rebecca Oyetoro, Maryam Hashemian, Alicia A. Livinski, Joseph J. Shearer, Jungnam Joo, Bruce M. Psaty, Daniel Levy, Peter Ganz, Véronique L. Roger

**Affiliations:** 1 Heart Disease Phenomics Laboratory, Epidemiology and Community Health Branch, National Heart, Lung, and Blood Institute, National Institutes of Health, Bethesda, MD USA; 2 Office of Research Services, Office of the Director, National Institutes of Health Library, National Institutes of Health, Bethesda, MD USA; 3 Office of Biostatistics Research, National Heart, Lung, and Blood Institute, National Institutes of Health, Bethesda, MD USA; 4 Cardiovascular Health Research Unit, Departments of Medicine, Epidemiology and Health Systems and Population Health, University of Washington, Seattle, WA USA; 5 Laboratory for Cardiovascular Epidemiology and Genomics, Population Sciences Branch, Division of Intramural Research, National Heart, Lung, and Blood Institute, National Institutes of Health, Bethesda, MD USA; 6 Zuckerberg San Francisco General Hospital, University of California, San Francisco, San Francisco, CA USA

**Keywords:** Proteomics, Heart failure, Aptamer-assay, Antibody assay, Systematic review, Mortality

## Abstract

**Background:**

Heart failure (HF) is a complex clinical syndrome with persistently high mortality. High-throughput proteomic technologies offer new opportunities to improve HF risk stratification, but their contribution remains to be clearly defined. We aimed to systematically review prognostic studies using high-throughput proteomics to identify protein signatures associated with HF mortality.

**Methods:**

We searched four databases and two clinical trial registries for articles published from 2012 to 2023. HF proteomics studies measuring high numbers of proteins using aptamer or antibody-based affinity platforms on human plasma or serum with outcomes of all-cause or cardiovascular death were included. Two reviewers independently screened articles, extracted data, and assessed the risk of bias. A third reviewer resolved conflicts. We assessed the risk of bias using the Risk Of Bias In Non-randomized Studies—of Exposure tool.

**Results:**

Out of 5131 unique articles identified, nine articles were included in the review. The nine studies were observational; three used the aptamer platform, and six used the antibody platform. We found considerable heterogeneity across studies in measurement panels, HF definitions, ejection fraction categorization, follow-up duration, and outcome definitions, and a lack of risk estimates for most protein associations. Hence, we proceeded with a systematic review rather than a meta-analysis. In two comparable aptamer studies in patients with HF with reduced ejection fraction, 21 proteins were identified in common for the association with all-cause death. Among these, one protein, WAP four-disulfide core domain protein 2 was also reported in an antibody study on HFrEF and for the association with CV death. We proposed standardized reporting criteria to facilitate the interpretation of future studies.

**Conclusions:**

In this systematic review of nine studies evaluating the association of proteomics with mortality in HF, we identified a limited number of proteins common across several studies. Heterogeneity across studies compromised drawing broad inferences, underscoring the importance of standardized approaches to reporting.

**Supplementary Information:**

The online version contains supplementary material available at 10.1186/s12916-024-03249-7.

## Introduction

Heart failure (HF) is a clinical syndrome characterized by persistently high morbidity and mortality, despite advances in medical management [1–3]. While some individuals with HF will require advanced therapies, including heart replacement, others will respond well to guideline-directed medical therapies. As stratifying the risk of HF clinically is challenging, more precise approaches to risk stratification are critically needed to guide clinical decision-making. This need is emphasized in the 2022 American Heart Association/ American College of Cardiology/Heart Failure Society of America guidelines, which underscore the promise of omics technologies for this purpose [4].

In the last decade, high-throughput proteomics technologies using affinity reagents have emerged that have the potential to respond to the stated need [5]. These affinity-based technologies rely on different methods to measure a large number of proteins: one method which currently targets approximately 7000 human proteins uses slow off-rate modified aptamers (SOMAmer) which are modified short, single-stranded oligonucleotides as protein-binding reagents which are quantifiable by nucleic acid microarrays [6, 7]. The second method uses an antibody-based proximity extension assay that has the capability of identifying up to nearly 3000 human proteins, by relying on the dual binding of antibodies to a target protein to minimize nonspecific binding, and cross-reactivity [8, 9]. Several studies applying these technologies have suggested their value for HF risk stratification [10–19]. The purpose of this study was to systematically identify, describe, and compare studies that used large-scale antibody or aptamer assays to identify protein biomarkers associated with all-cause or cardiovascular (CV) death in HF. In doing so, we highlight important methodological elements to offer recommendations for future reporting.

## Methods

This systematic review was written following the Preferred Reporting Items for Systematic Reviews and Meta-analyses (PRISMA) checklist [20]. The PRISMA Protocol extension [21] was used for writing the protocol a priori, which was registered in the PROSPERO (identifier: CRD42023449663).

### Eligibility criteria (Table 1)

**Table 1:** Eligibility criteria for study selection

Category	Inclusion criteria	Exclusion criteria
Population	• Human • Adults (aged ≥ 18 years) with HF irrespective of EF	• Non-human studies • Children and adolescents (aged 2–17 years) • Adults without HF
Measurement(s)/exposure(s)	• Assessed the plasma or serum proteome • Proteins measured by high-throughput affinity-based proteomic techniques including aptamer-based (SomaScan) or antibody-based (Olink)	• Assessed biological proteome other than plasma or serum • Proteomic techniques other than the large-scale affinity platforms
Comparator/association(s)	• Reported protein association with outcomes	• No protein association with outcomes reported
Outcome(s)	• All-cause death • Cardiovascular death • A composite outcome that includes death	• Absence of death outcomes
Study design(s)	• Observational studies (all types, e.g., cohort, case–control, cross-sections, longitudinal, prospective, retrospective) • Clinical trials	• Articles without primary data (commentaries, editorials, protocols, letters, reviews of all types) • Incomplete data (conference abstracts/proceedings)
Language	• No restrictions	• NA
Date of publication	• 2012 to June 2023	• Before 2012

*HF* Heart failure, *EF* Ejection fraction

We included observational studies and all phases of clinical trials of (1) adults (aged ≥ 18 years) diagnosed with prevalent or incident HF with reduced ejection fraction (HFrEF) or preserved ejection fraction (HFpEF), (2) with plasma or serum proteome measures from aptamer-based (SomaScan) or antibody-based (Olink) high-affinity proteomic assays, (3) focused on protein association with outcomes, (4) reporting outcomes including all-cause death, CV death, or a composite outcome that includes death, and (5) published after 2012.

### Information sources and search strategy

A biomedical librarian (AAL) searched four databases: Embase (Elsevier), PubMed (US National Library of Medicine), Scopus (Elsevier), and Web of Science: Core Collection (Clarivate Analytics) in March 2023, for proteomic studies in patients with HF published since 2012. Additionally, two clinical trial registries, Cochrane Library’s CENTRAL database (Wiley & Sons) and ClinicalTrials.gov (US National Library of Medicine) were searched in May 2023 for HF proteomic studies. EndNote 20 (Clarivate Analytics) was used to collect all records and identify duplicates.

The search strategies used are shown in Additional file 1. No publication language restrictions were used.

### Selection process

First, a pilot of the two-step screening process using a random sample of 30 articles was completed by two reviewers (KOK and RO) using Covidence systematic review software (Veritas Health Innovation, Melbourne, Australia).

The two-step screening process was conducted using Covidence. For screening, the titles and abstracts of all unique records from the database searches were independently screened in duplicate by two reviewers (KOK and RO) using the established eligibility criteria (Table 1). Any conflicts or disagreements were resolved by discussion between the reviewers.

Next, the full-text screening of the records included after the first step was performed by two reviewers (KOK and RO) independently and in duplicate using the eligibility criteria. For this step, any conflicts were resolved by a third reviewer (MH).

### Data collection and data items

For data collection, we created a standardized table of data items and definitions in Microsoft Excel. Two reviewers (KOK and RO) independently collected the data from each included article. The extracted items were verified by a third reviewer (MH) who resolved any discrepancies in the collected data. For each included study, we collected first author name, publication year, study design, name of cohort/registry, number of participants, years of enrollment, demographics (age, sex, and race/ethnicity or ancestry), ejection fraction (EF) category and definition, assay information (panel version and number of targets), outcome(s), and key findings on the number of significant proteins associated with outcomes (all-cause, CV death, and composite outcomes including death).

### Risk of bias assessment

Given the observational nature of the studies included, we assessed the risk of bias for individual studies using the Risk of Bias for Non-randomized Studies—of Exposures (ROBINS-E) tool [22]. Two reviewers (KOK, RO) independently completed the assessment of the included articles, and a third reviewer (MH) checked the results and helped achieve consensus when there was disagreement on the assessed level of risk. Seven domains were covered in the ROBINS-E tool to evaluate bias due to (1) confounding, (2) exposure classification, (3) selection of study participants, (4) departures from intended exposures or post-exposure intervention, (5) missing data, (6) outcome measurement, and (7) the selection of reported results. Each domain was characterized as having low, moderate (some concerns), and high risk of bias. After completing all seven bias domains, an overall assessment was derived from the domain-level judgments using the ROBINS-E tool.

Our evaluation of protein biomarkers, outcome data, and study comparisons was limited to articles assessed with a low or moderate risk of bias.

### Data synthesis

We reviewed proteomic associations with all-cause, cardiovascular death, or any composite endpoint that included death as one of the elements of the composite event. We did not consider hospitalizations, which are challenging to interpret due to their inherently multifactorial nature including but not limited to worsening HF, other comorbidities, but also access to care and its multiple determinants [23]. Protein lists from the aptamer and antibody platforms were confirmed using the information on the manufacturers’ websites, (www.somalogic.com and www.olinkexplore.com), in addition to published lists [24]. Due to variations in nomenclatures to designate specific proteins, we used UniProt.IDs [25] to compare findings across studies (www.uniprot.org) and to identify the common proteins. We sought the availability of risk estimates for individual proteins in manuscript tables, figures, texts, and supplemental material. For the studies reporting both minimally and fully adjusted models, only the results from fully adjusted models were considered. To characterize the functional classes of the proteins considered, we relied on the PANTHER Protein Class ontology (http://www.pantherdb.org/) [26].

## Results

### Selection of studies

**Fig. 1:**
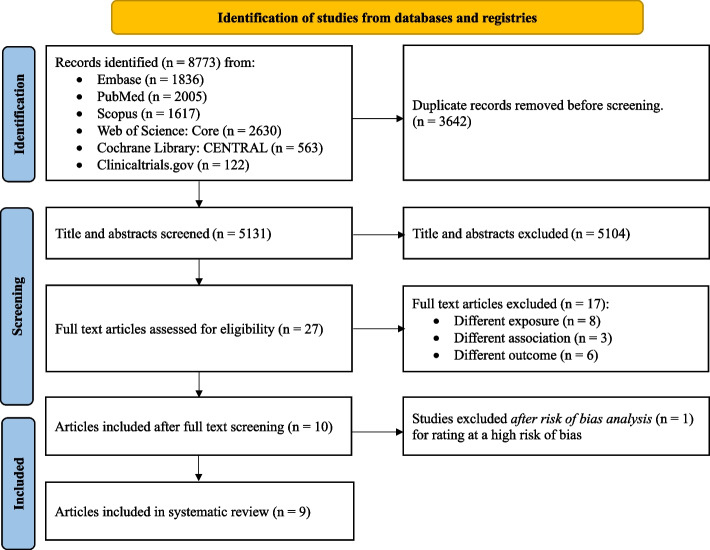
The PRISMA flow diagram

As summarized in the PRISMA diagram (Fig. 1), the database and registry searches retrieved 8773 articles of which 3642 were duplicates and 5131 were screened at title and abstract. Of the 5131, we excluded 5104 articles, leaving 27 for full-text screening. After completing the full-text screening, we excluded 17 articles leaving 10 eligible articles, which we then assessed for risk of bias [10, 11, 13, 15, 16, 27–31]. One of the 10 articles was excluded after the risk of bias assessment (Additional file 2: Fig. S1). Therefore, nine articles published between 2017 and 2022 [10, 11, 13, 15, 16, 27–30] were included in this systematic review (Table 2).

**Table 2:** Characteristics of included studies

First author, year	Design	Population(s)	Enrollment	Demographics	Ejection fraction	Assay	Outcome(s)	Key findings
Zhang[10], 2022	Secondary analysis of a clinical trial	Derivation cohort: ATMOSPHERE trial ^31^ (*n* = 1258)	2009–2013	Median age: 67 (59;73), 19% female, 96% White	HFrEF (≤ 35%)	SomaScan 5 K version 3 (4076 unique proteins 5034 SOMAmers)	Primary: Composite of CV death or first HFH; Secondary: all-cause death, CV death, HFH	• 64 proteins associated with composite outcomes replicated in the validation cohort • 105 proteins associated with all-cause death • 80 proteins associated with CV death
		Validation cohort: PARADIGM-HF trial ^32^ (*n* = 1257)	2009–2013	Median age: 68 (61;74), 19% female, 96% White				
Gui [13], 2021	Observational HF Registry	Derivation cohort: Henry Ford (*n* = 681)	2007–2015	Mean age: 67.9 ± 11.5, 36% female, 50.4% White	HFrEF (< 50%)	SomaScan 5 K version 4 (4111 unique proteins; 4453 SOMAmers)	Primary: All-cause death; Secondary: CV death	• 128 proteins associated with all-cause death • No report on individual proteins associated with CV death
		Validation cohort: Henry Ford (*n* = 336)		Mean age: 67.8 ± 12.1, 33.6% female, 48.8% White				
Cuvelliez [15], 2019	Observational cohort (nested case–control)	INCA Study Population (*n* = 168)	1998–2001	Participants with composite outcome (*n* = 84): Mean age: 59 ± 11, 12% female	HFrEF (≤ 45%)	SomaScan 1.3 K version 1.2 (1305 proteins; 1310 SOMAmers)	Composite (CV death, urgent transplant, urgent assist device implantation)	• 203 proteins expressed differently between patients who died of CV causes (composite) and the patients who were alive. •
				Participants without composite outcome (*n* = 84); Mean age: 59 ± 10, 12% female				
Klimczak-Tomaniak [28], 2022	Observational cohort	Bio-SHiFT Study Population (*n* = 250)	2011–2013	Participants with composite outcome (*n* = 66): Mean age: 71.4 ± 19, 21% female	HFrEF (< 50%)	Olink Proteomics: Panel CVIII; 92 serially measured proteins	Composite (cardiac death, transplant, LVAD implantation, acute or worsened HF)	• 73 proteins associated with a composite outcome including cardiac death • The optimal set of biomarkers selected by LASSO included 9 proteins
				Participants without composite outcome (*n* = 184): Mean age: 66.2 ± 16, 28% female				
Markousis-Mavrogenis [29], 2022	Observational cohort	Index cohort: BIOSTAT-CHF cohort (*n* = 2022)	2010–2012	Mean age: 68.8 ± 12, 26.6% female	HFrEF (≤ 40%) HFpEF (≥ 50%)	Olink Proteomics Multiplex (Panels CVII, CVIII, Immune Response, and Oncology-II); 355 proteins	All-cause death	• 187 immune-related proteins associated with all-cause death in univariate analyses
		Validation cohort: BIOSTAT-CHF cohort (*n* = 1691)		Mean age: 73.7 ± 10.7, 24.1% female (based on cohort [*n* = 1738])				
Ravera [30], 2022	Observational cohort	Index cohort: BIOSTAT-CHF cohort (*n* = 2022)	2010–2012	Women group (*n* = 537): Mean age: 71 ± 12 Men group (*n* = 1485) Mean age: 67 ± 12 27% female	HFrEF (≤ 40%) HFpEF (≥ 50%)	Olink Proteomics Multiplex (Panels CVDII, CVDIII, Immune Response, and Oncology-II panels); 363 proteins	All-cause death	• 8 and 12 proteins associated with all-cause death in the index and validation cohort respectively in both men and women
		Validation cohort: BIOSTAT-CHF cohort (*n* = 1698)		Women group (*n* = 575): Mean age: 74 ± 11 Men group (*n* = 1123): Mean age: 73 ± 11, 34% female				
Regan [11], 2022	Observational	CATHGEN discovery cohort (*n* = 176, non-HF: 88; HFpEF: 88)	2001–2010	Non-HF: Mean age: 53.1 ± 12.3, 42% female HFpEF: Mean age: 64.7 ± 12.3, 42% female	HFpEF (≥ 45%)	Olink 1200 Proteomics (Panels CVDII, CVDIII, Cardiometabolic, Metabolism and Development; 459 proteins	HFH and all-cause death	• 11 proteins associated with all-cause death across the 3 cohorts
		TECOS validation cohort (*n* = 109, non-HF:79; HFpEF: 30)	2008–2015	Non-HF: Mean age: 65.6 ± 8.5, 20.2% female HFpEF: Mean age:64.5 ± 8.3, 36.7% female	HFpEF (≥ 55%)			
		Jackson Heart Study cohort (all-cause death: 570; HFH: 448)	2005–2015	all-cause death: Mean age: 59 ± 12, 59% female HFH: Mean age: 58 ± 13, 59% female	HFpEF (≥ 50%)	Olink 1500 (Panels Cardiometabolic, CVDII, CVDIII, Cell regulation, Development, Immune, Immuno-Oncology-II, Inflammation, Metabolism, Neurology, Neuro-exploratory, Oncology II, Oncology III, Organ Damage); 369 proteins	Primary: HFH Secondary: all-cause death	
Ferreira [27], 2021	Observational	Index: BIOSTAT-CHF cohort (*n* = 1611)	2010–2012	< 65 years age group Mean age: 55 ± 8, 18.1% female 65–75 years age group Mean age: 70 ± 3, 20.4% female > 75 years age group Mean age: 81 ± 4, 37.3% female	HFrEF (≤ 40%)	Olink technology (Panels CVDII, CVDIII, immune response, and immuno-oncology panels); 363 proteins	Primary: Composite of HFH and all-cause death Secondary: All-cause death CV death non-CV death	• 11 proteins independently associated with age and all-cause death • 6 proteins associated with age and CV death after cross-validation
		Independent validation: BIOSTAT-CHF cohort (*n* = 823)		< 65 years age group Mean age: 57 ± 7, 27.6% female 65–75 years age group Mean age: 70 ± 3; 20.4% Female > 75 years age group Mean age, 81 ± 4, 37.3% female				
Hage [16], 2017	Observational	KaRen study biomarker substudy, no outcome group (*n* = 50)	2007–2011	ALL: Median age: 73 (66; 78) 51% female No outcome group: 73 (66; 81) 48%; female Outcome group: 74 (66; 79) 57% female	HFpEF (≥ 45%)	Olink technology Panel CVD I v1; 92 proteins	Composite (HFH or all-cause death)	• 28 biomarkers associated with composite outcome
		KaRen study biomarker substudy, outcome group (*n* = 36)						

*CV* Cardiovascular, *HF* Heart failure, *HFrEF* Heart failure with reduced ejection fraction, *HFH* Heart failure hospitalization, *HFpEF* Heart failure with preserved ejection fraction, *SOMAmers* Slow Off-rate Modified Aptamers

### Characteristics of included studies

All studies were observational including seven prospective cohorts [13, 15, 16, 27–30], one using two clinical trial populations [10], and the last study [11] included three groups with different designs: two nested case–control designs [32, 33], and a population-based cohort [34]. The studies were conducted in the USA [13], France [15], the Netherlands [28], Sweden [16], and three multi-national studies including participants mostly from Europe, the Americas, and Asia [10, 11, 27]. Notably, three studies included patients from the same cohorts [27, 29, 30]. While there were large variations in sample size, we enumerated a total of 7773 participants across the studies; their ages ranged between 53 and 71 years and 41% of participants were women. Two studies that reported race included 96% and 50% patients of European ancestry, respectively [10, 13]. Six of the studies included both derivation and validation or replication cohorts [10, 11, 13, 27, 29, 30].

### Clinical characteristics, proteomics measurements, and outcomes

The use of HF diagnostic criteria was reported in three studies [13, 16, 28], two cited the Framingham criteria [13, 28] and one study cited the European Society Guidelines [16] criteria. Five of the studies focused on patients with HFrEF, two studies focused on patients with HFpEF, and two studies included the entire spectrum of HF regardless of EF categorization [29, 30]. The follow-up ranged from 9 to 60 months. Three studies used aptamer assays (*n* = 1310–4111) and six used antibody assays (*n* = 92–459). Hence, the heterogeneity across studies was quite substantial pertaining to the assays used, definitions of HF, categorization of EF, follow-up duration, outcomes selection, and definitions. This precluded the conduct of a meta-analysis leading us to proceed with the following systematic review.

### Studies by platform

#### Aptamer-based studies

The three aptamer studies were restricted to HFrEF defined by different cut points (EF ≤ 35%, EF < 50%, and EF ≤ 45%) [10, 13, 15]. Two studies [13, 15] used plasma and one serum [10]. Two of the studies used the SomaScan 5k platform (versions 3 and 4) [10, 13] while an earlier version (SomaScan Assay 1.3K. version 1.2) was used in the third study (Table 2) [15].

All three studies developed models using the Least Absolute Shrinkage and Selection Operator (LASSO) penalized regression. The genetic association of specific protein targets was evaluated in two of the studies using protein quantitative trait loci (pQTL) sources [35] to assess aptamer specificity [10, 13].

#### Antibody-based studies

Among the six antibody-assay studies, two studied HFrEF [27, 28], two HFpEF [11, 16], and two included both HFrEF and HFpEF. The EF group cut points also varied across the studies (Table 2). One of the HFpEF studies included three different groups with different HFpEF cutoffs (EF ≥ 45%, EF ≥ 55%, and EF > 50%) [11]. All six studies applied a limited number of panels available in Olink® targeted panels ranging from 92 to 459 proteins (Table 2).

### Associations with outcomes

**Fig. 2:**
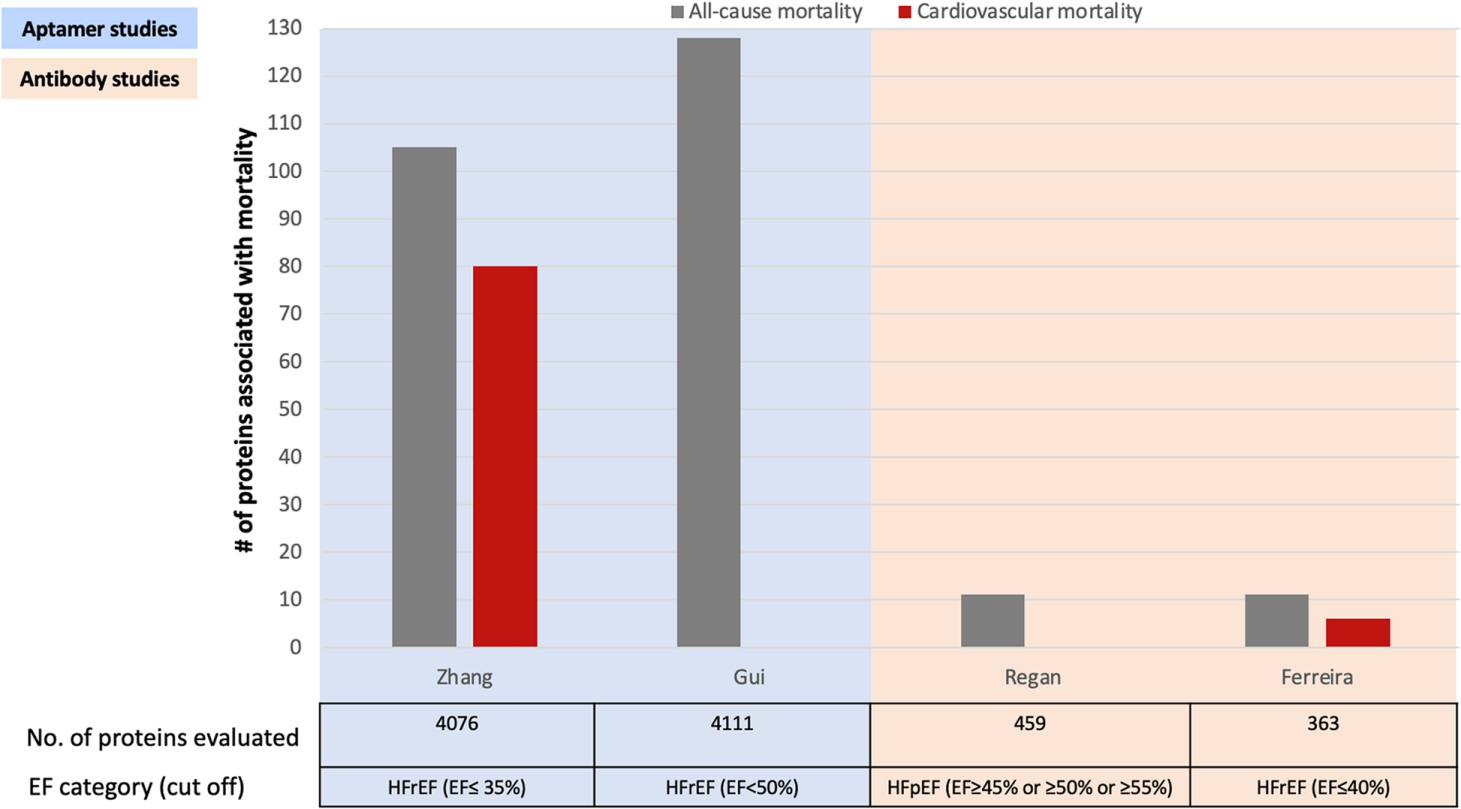
Number of proteins associated with death

Among the nine studies, five studies were not considered further (three studies reported only composite outcomes with varied components [15, 28, 36], one focused on the association with the outcome for proteins differentially expressed by sex and did not report overall results [30], and one was restricted to the exploration of immune-related mechanisms) [29]. Thus, we were left with four studies that reported associations with all-cause death [10, 11, 13, 27] among which two also reported on CV death [10, 27] (Fig. 2).

#### All-cause death

Two aptamer assay studies had reasonably comparable design features: restriction to HFrEF, and report of all-cause death, despite differences in panel version, HFrEF cut-offs, and model adjustment [10, 13]. Comparing the two studies [10, 13], one reported 84 and the other reported 107 unique proteins associated with all-cause death, and 21 proteins were identified in common between the studies [10, 13] (Table 3). Risk estimates with confidence intervals were not provided precluding a meta-analysis of the common proteins between these two studies. The functional class of the common proteins based on their encoded gene by PANTHER Protein Class ontology are listed in Table 3.

**Table 3:** UniProt accession number, name, and class of 21 proteins associated with all-cause death in HF and identified by two studies on aptamer platforms (online sources: UniProt website (UniProt) and PANTHER (http://www.pantherdb.org/)

UniProt accession	Protein name	PANTHER protein class
P61769	b2-Microglobulin	Major histocompatibility complex
Q86VB7	Scavenger receptor cysteine-rich type 1 protein M130	Serine protease
P09326	CD48 antigen	Immunoglobulin receptor superfamily
P39060	Collagen alpha-1 (XVIII) chain	Extracellular matrix structural protein
Q2UY09	Collagen alpha-1(XXVIII) chain	Extracellular matrix structural protein
P12111	Collagen alpha- 3(VI) chain	Extracellular matrix structural protein
P01034	Cystatin-C	Protease inhibitor
P29317	Ephrin type-A receptor 2	Protease inhibitor
O95633	Follistatin-related protein 3	Protease inhibitor
Q9UJJ9^a^	N-acetylglucosamine-1-phosphotransferase subunit gamma	Protein-binding activity modulator
P28799	Progranulin	No class assigned
Q14508	WAP four-disulfide core domain protein 2	Protease inhibitor
P01861^a^	Immunoglobulin heavy constant gamma 4	Immunoglobulin receptor superfamily
Q13261	Interleukin-15 receptor subunit alpha	Transmembrane signal receptor
P22897	Macrophage mannose receptor 1	Membrane trafficking regulatory protein
Q93091	Ribonuclease K6	Endoribonuclease
P07998	Ribonuclease pancreatic	Endoribonuclease
Q03403	Trefoil factor 2	Intercellular signal molecule
Q07654	Trefoil factor 3	Intercellular signal molecule
P19438	Tumor necrosis factor receptor superfamily member 1A	Transmembrane signal receptor
P20333	Tumor necrosis factor receptor superfamily member 1B	Transmembrane signal receptor

^a^Proteins not detectable by the antibody assay (Olink® Explore 3072) applied in included studies. All others are detectable. (www.olinkexplore.com)

One antibody assay study on HFrEF also had a relatively comparable design to the two aptamer HFrEF studies and reported five unique proteins to be associated with all-cause death [27]. A single protein, WAP four-disulfide core domain protein 2 (also known as human epididymis protein 4 (HE4)) was identified in all three HFrEF studies for the association with all-cause death [10, 13, 27]. In addition, five proteins (R-spondin3 (RSPO3), triggering receptor expressed on myeloid cells (TREM1), C-X-C motif chemokine receptor (CXCL13), osteoprotegerin (OPG), and stem cell factor (SCF)) partially overlapped between the studies.

#### Cardiovascular deaths

Only two studies reported associations of proteins with CV death in HFrEF, one on each platform [10, 27]. In the aptamer study, 77 unique proteins were identified [10] while the antibody study reported three unique proteins [27] for the association with CV death (Fig. 2). The two studies shared three proteins: RSPO3, TREM1, and WAP four-disulfide core domain protein 2.

### Overlap across EF groups

Few proteins were reported in common for all-cause death across studies in the different EF groups: HFrEF [10, 13, 27] and HFpEF [11]. Two antibody studies, one in HFpEF (Regan et al.) [11] and the other in HFrEF (Ferreira et al.) [27] each found 11 proteins associated with all-cause death and reported one protein (prolargin — an extracellular matrix protein) in common [11, 27]. Also, one study in HEpEF (Regan et al.) [11] reported one protein (vascular endothelial growth factor D — involved in angiogenesis and remodeling) in common with the other study in HFrEF (Zhang, et al.) [10] for the association with all-cause death.

### Studies with risk scores

Five studies developed multi-protein scores to predict all-cause death or composite outcomes and examined the incremental value of these scores over clinical data, most frequently represented by the MAGGIC score and NTproBNP [10, 11, 13, 15, 28]. While the incremental value of the multiprotein scores varied across studies, their comparison is compromised by the degree of heterogeneity across studies which is compounded by differences in the adjusted models applied in generating the scores.

## Discussion

To our knowledge, this is the first systematic review of proteomic studies using affinity reagents evaluating death in HF. We selected nine studies based on our inclusion criteria and risk of bias assessment [22]. The studies were highly heterogeneous, with respect to definitions of HF, choices of EF cut-points, assay methods, coverage of the proteome, follow-up duration, and outcomes reported. This heterogeneity precluded the conduct of a meta-analysis, leading us to conduct a systematic review.

Three of the studies (two aptamer- and one antibody-based) reported on all-cause death in HFrEF [10, 13, 27]. Twenty-one proteins were identified in common by the aptamer studies. One of these, WAP four-disulfide core domain protein 2 was associated with all-cause death in all three HFrEF studies [10, 13, 27]. Furthermore, WAP was reported for the association with CV death in two of the three studies [10, 27]. Due to methodological differences including variations in proteomic measurement assay and in the outcomes reported, the studies focused on HFpEF could not be compared with one another.

### The proteomic platforms

Three studies [10, 13, 15] utilized the SomaScan aptamer platform which is reported as having a wider human proteome coverage [37, 38]. Six studies [11, 16, 27, 28, 30, 31] used Olink antibody-based assay which is reported to have stronger protein target specificity based on the percentage of proteins on the platform with reported genetic association [37]. However, all six antibody-based studies used panels containing only a subset (3–15%) of the 3072 Olink Explore panel while aptamer studies reported the full array of proteins available on their respective SomaScan versions. Consequently, only 13 of the 21 common proteins between the qualitatively comparable aptamer studies would have been detectable by the antibody studies in this review. Therefore, it is conceivable that a greater number of overlapping proteins might have been identified across the platforms if complete panels were used in the antibody studies. Overall, due to the evolving landscape of both aptamer and antibody proteomic assays with respect to coverage, sensitivity, and validation [37, 39], platform selection considerations in different studies warrant further studies.

### Common findings across studies

Two aptamer-based HFrEF studies that reported on all-cause death identified 21 common proteins. One of these proteins, WAP four-disulfide core domain protein 2 was also associated with all-cause death in an antibody study and in the two studies that examined CV death [10, 13, 27]. Hence, WAP four-disulfide core domain protein 2 emerged as a protein of interest for risk stratification in HF, at least when the EF is reduced.

WAP four-disulfide core domain protein 2 is a protease inhibitor with roles in innate immunity and tumorigenesis. Clinically, it has been well studied as a novel therapeutic marker of epithelial ovarian and endometrial cancer [40–42]. More recently WAP was shown to be associated with growth differentiation factor 15 (GDF15) levels, which is expressed in inflammation and myocardial ischemia [43] and linked with poor outcomes in HF [44–46]. In a clinical trial sub-study of over 500 patients, WAP four-disulfide core domain protein 2 was associated with HF severity and the composite outcome of all-cause death or HF hospitalization and improved risk stratification over common clinical markers [47]. The present systematic review amplifies the findings from these prior reports and calls for additional studies evaluating WAP as a biomarker across the entire spectrum of HF syndrome.

Other proteins were found in common across the HFrEF studies on the different platforms including RSPO3 and OPG, which are both involved in fibrosis, and OPG has been previously reported as associated with HF prognosis [48, 49]. TREM1 and CXCL13 also in common between two of the HFrEF studies across platforms are both inflammatory proteins that have been linked to cardiac remodeling. CXCL13 is believed to be regulated in HF and atherosclerotic lesions alongside its receptor (C-X-C motif chemokine receptor 5-CXCR5) [50–52]. Lastly, SCF is a hematopoietic cytokine that may have a role in ischemia [53].

### Heterogeneity across studies

In addition to differences in the coverage of the proteome related to differences in platforms, we observed considerable heterogeneity in methodology across the studies. With respect to HF diagnosis, only 3 studies specified their choice of criteria and used two different definitions [13, 16, 28]. The prevalence and case mix of HF varies depending on diagnostic criteria underscoring the need for caution in comparing results across studies [2, 54, 55]. Cut-offs selected for categorization into HFrEF or HFpEF were equally heterogenous: five HFrEF studies had four different cutoffs, and one of the two HFpEF studies included three cohorts with different EF cutoffs. Though EF provides a basis for clinical HF classification, its relevance to the study of proteomics is not clear. One cross-sectional study suggested differences in the circulating proteome across EF groups [56], but the effect of these differences on death is uncertain, particularly given the variability across EF categories in the literature. Few proteins overlapped across studies restricted to HFrEF or HFpEF [10, 11, 13, 15, 16, 27, 28], suggesting some commonality of prognostic value [56]. The duration of follow-up varied across different studies.

Also, the study outcomes were heterogeneous. Six of the nine studies focused on all-cause and/or CV death [10, 11, 13, 27, 29, 30], while the remaining reported composite outcomes that included death however defined differently across the studies [15, 16, 28]. Composite outcomes are commonly used in clinical trials to increase the number of events and improve study power, but improvements in power are contingent upon similar direction and magnitude of risk associated with the individual components of the composite outcome [57, 58].

### Common pitfalls across studies

Clinical research studies can serve two distinct purposes: prediction and etiology. Prediction studies primarily aim for risk stratification, offering valuable insights into the likelihood of specific outcomes. However, prediction equations do not inherently provide insights into biological mechanisms or novel therapeutic approaches. In prediction-focused studies, the impact of confounding variables is generally less relevant [59, 60].

On the other hand, clinical research studies can uncover risk factors, unravel underlying biological processes, and potentially unveil new targets for therapeutic interventions. In these etiologic studies, accounting for confounding variables becomes crucial [59]. Often, the findings from such proteomic studies are further examined using Mendelian randomization analyses, which can provide evidence of a potential causal relationship.

It is essential to distinguish between these two categories in proteomics studies — prediction and etiology — when presenting research results. Keeping these purposes separate helps maintain the integrity of study designs and analytical methodologies. In this review, we observed that majority of the studies combined these two purposes.

Additionally, the use of inception cohorts of newly diagnosed HF cases is the preferred design for both prediction and etiologic studies [61]. Cohort studies of prevalent cases are vulnerable to survival bias [62].

### Recommendations for future reports

The widespread interpretation challenges discussed above led us to formulate reporting recommendations to facilitate the interpretation of future studies. Several of them are focused on the adoption of state-of-the-art methods for the design and analysis of observational studies. These standards are not new, but their importance cannot be overemphasized as it is critical to abide by them so that the findings of proteomics studies can be compared, and the data pooled for group or individual-level meta-analyses.

- *Standard reporting guidelines* such as the STROBE statement [63] (for observational studies), and TRIPOD statement [64] (for risk prediction studies) should be used to ensure complete reporting and will facilitate the assessment of studies’ strengths and weaknesses [65].
- *Study goals* should be explicitly defined as either a prediction study or an etiology study and the analysis should be designed in accordance with stated goals.
- *Design* should be that of inception-cohort whereby all subjects are enrolled at the same disease stage to the extent possible [61]. Attention to the possibility of index-event bias is also important [66].
- *Recruitment strategies* should be explicitly designed to ensure the enrollment of diverse populations in sufficient numbers to enable analyses stratified by race/ethnicity and sex.
- The *ascertainment of HF* should rely on standardized criteria, such as the Framingham criteria [67] or the European Society Guidelines [68].
- *Ejection fraction categories*: Studies should include all forms of the HF syndrome including the entire EF spectrum. EF categories should be defined using cut-points recommended by the HF guidelines while however acknowledging the lack of consensus across guidelines [4, 68, 69]. The data should be analyzed while including all patients followed by stratified analyses by EF categories and sensitivity analyses to account for variability in EF cut points.
- *Study endpoints* should include all-cause death and CV death. When composite outcomes are used individual analyses of the components of said composite outcomes should be reported.
- *Approaches to validation*: the findings obtained in derivation cohorts should be validated. This could be accomplished using internal validation or external validation [70]. In the absence of an external validation cohort, temporal validation can be used as an alternative method as indicated in the TRIPOD guidelines. Orthogonal validation of identified proteins is also possible through several means including data from mass spectrometry [71, 72] and genome-wide association studies [37, 73]. The integration of population genomics with high-throughput proteomics can strengthen orthogonal validation and comparisons of identified proteins [39, 74], thereby enhancing the understanding of the correlations and differences among proteins measured on various platforms.
- *Protein nomenclature:* reliance on UniProt.IDs [25] in addition to protein target names to facilitate comparison across studies.
- Populations at high risk of adverse outcomes benefit from *near-term* risk prediction and risk models should be designed to provide this information as well as longer-term time horizons [75].

## Strengths and limitations

This review has important strengths. First, we designed a comprehensive and rigorous search strategy to capture prognostic HF studies using high-throughput proteomics. Second, we assessed the risk of bias to guide our selection of the studies considered and omitted from our analyses the study assessed at a high risk of bias.

This systematic review was limited by the heterogeneity of the studies in addition to the non-availability of hazard ratios and confidence intervals in some studies which precluded the performing a meta-analysis.

## Conclusions

We performed a systematic review evaluating the literature on high-throughput proteomics using affinity reagents to characterize proteins associated with death outcomes in patients with HF. Though we report overlapping proteins for all-cause death in HFrEF studies and singled out markers for future studies, the methodological differences noted call for caution in the aggregate interpretation of the findings. Our review points to the substantial heterogeneity across HF prognostic studies using high-throughput proteomic assays, which constitutes a strong rationale to adopt standardized recommendations to strengthen future studies on this topic.

### Supplementary Information

**Additional file 1.** Search Strategy.

**Additional file 2.** Risk of bias assessment using the ROBINS-E tool.

## Data Availability

Materials used in this review are available in the reports and the databases used for this review.

## Abbreviations

HF: Heart failure
HFrEF: Heart failure with reduced ejection fraction
HFpEF: Heart failure with preserved ejection fraction
CV: Cardiovascular
PRISMA: Preferred Reporting Items for Systematic Reviews and Meta-analyses
EF: Ejection fraction
SOMAmer: Slow off-rate modified aptamers.
MAGGIC: Meta-Analysis Global Group in Chronic Heart Failure
PROSPERO: International Prospective Register of Systematic Reviews
ROBINS-E: Risk of Bias for Non-randomized Studies—of Exposures
HFH: Heart failure hospitalization
LASSO: Least Absolute Shrinkage and Selection Operator
pQTLs: Protein quantitative trait loci
