# Supplementary material for: Proteomics for heart failure risk stratification: a systematic review
Source: BMC Med. 2024 Jan 25;22:34. doi: 10.1186/s12916-024-03249-7 (PMC10809595; doi:10.1186/s12916-024-03249-7)
Supplement: Supplementary file 1 — Additional file 1. Search Strategy. [file 12916_2024_3249_MOESM1_ESM.docx]

**Proteomics for Heart Failure Risk Stratification: A Systematic Review**

**Search Strategy**

The search strategy was initially developed by the biomedical librarian and reviewed by members of the review team for input and feedback. Further revisions were made, and another biomedical librarian not affiliated with this review informally reviewed the initial search strategy. The biomedical librarian was responsible for developing the initial draft, soliciting, reviewing, and incorporating feedback, and running and adapting the search strategy for each database searched. The complete search strategy is shown below.

**Final Search Strategies Used**

**Database:** PubMed
**Platform:** US National Library of Medicine
**Date Searched:** March 1, 2023
**Date Limits:** January 1, 2012 – December 31, 2022
**Other Limits/Filters:** A search strategy was used to remove animal studies (i.e., non-human) and specific article types (e.g., conference abstracts, letters, editorials, reviews) that were detailed in our exclusion criteria from the search results).

| **Set** | **Concept** | **Search Strategy** |
| --- | --- | --- |
| #1 | Proteomics | (“Proteomics”[Mesh] OR “Proteome”[Mesh] OR proteom*[tiab] OR “aptamer based”[tiab] OR “antibody based”[tiab] OR “aptamer-based”[tiab] OR “antibody-based”[tiab] OR somalogic[tiab] OR Luminex[tiab] OR Olink[tiab] OR “o link”[tiab] OR ((biomarker*[tiab] OR “biological marker*”[tiab] OR “biologic marker*”[tiab] OR “biochemical marker*”[tiab] OR Biomarkers[Mesh:noexp]) AND (protein[tiab] OR proteins[tiab] OR “Proteins”[Mesh:NoExp])) OR “serum marker”[tiab] OR “serum markers”[tiab] OR “serum proteome*”[tiab] OR “serum proteomics”[tiab] OR “serum biomarkers”[tiab] OR “serum biomarker”[tiab] OR “plasma proteome*”[tiab] OR “plasma proteomics”[tiab] OR “plasma marker”[tiab] OR “plasma markers”[tiab] OR “plasma biomarker”[tiab] OR “plasma biomarkers”[tiab] OR “prote-omic*”[tiab] OR “protein omic*”[tiab] OR proteinomic*[tiab]) |
| #2 | Heart failure | (“Heart Failure”[Mesh:noexp] OR “Heart Failure, Diastolic”[Mesh] OR “Heart Failure, Systolic”[Mesh] OR “heart failure*”[tiab] OR “cardiac failure*”[tiab] OR “myocardial failure*”[tiab] OR “systolic failure*”[tiab] OR “diastolic failure*”[tiab] OR HfrEF[tiab] OR HfpEF[tiab] OR HfmrEF[tiab] OR “diastolic dysfunction*”[tiab] OR “systolic dysfunction*”[tiab] OR cardiomyopath*[tiab] OR “cardiac decompensation*”[tiab] OR “cardiac incompetenc*”[tiab] OR “cardiac insufficienc*”[tiab] OR “cardial decompensation*”[tiab] OR “cardial insufficienc*”[tiab] OR “chronic heart insufficienc*”[tiab] OR “heart decompensation*”[tiab] OR “heart insufficienc*”[tiab] OR “myocardial insufficienc*”[tiab]) |
| #3 |  | #1 AND #2 |
| #4 | Limits: | #3 AND (“2012”[Date – Publication] : “2022”[Date – Publication]) |
| #5 |  | #4 NOT (“Animals”[Mesh] NOT (“Animals”[Mesh] AND “Humans”[Mesh])) NOT (mice[tiab] OR mouse[tiab] OR murine*[tiab] OR rat[tiab] OR rats[tiab] OR rodent*[tiab] OR dog[tiab] OR dogs[tiab] OR pig[tiab] OR pigs[tiab] OR piglet*[tiab] OR swine[tiab] OR porcine*[tiab] OR animal*[tiab] OR ape[tiab] OR apes[tiab] OR monkey*[tiab] OR gorilla*[tiab] OR chimpanzee*[tiab] OR macaque*[tiab] OR orangutan*[tiab] OR pongo[tiab] OR macaca[tiab] OR Muridae[mesh] OR Mice[mesh] OR Rats[mesh] OR “Pan paniscus”[Mesh] OR “Pongo”[Mesh] OR “Macaca”[Mesh] OR “Gorilla gorilla”[Mesh] OR Dogs[mesh] OR Swine[mesh] OR Rodentia[mesh] OR “Models, Animal”[Mesh] OR “Animal Experimentation”[Mesh]) |
| #6 |  | #5 NOT (editorial[Publication Type] OR comment[Publication Type] OR news[Publication Type] OR “Congress”[Publication Type] OR “Consensus Development Conference”[Publication Type] OR editorial[tiab] OR commentary[tiab] OR “conference abstract*”[tiab] OR “conference proceeding*”[tiab] OR symposium*[tiab] OR “Published Erratum”[Publication Type] OR errata[tiab] OR erratum[tiab] OR corrigenda[tiab] OR corrigendum[tiab] OR protocol[ti] OR protocols[ti] OR “Review”[Publication Type] OR “systematic review*”[tiab] OR “Systematic Review”[Publication Type] OR “integrative review*”[tiab]) |

**Notes:** Search field tags used in the above search.

- [Mesh] = [Medical Subject Heading (MeSH)](https://www.nlm.nih.gov/mesh/meshhome.html) with automatic explosion to include other MeSH terms below it in hierarchy unless otherwise specified as [Mesh:noexp];
- [tiab] = term(s) in title, collection title, abstract, other abstract, or keywords;
- [ti] = term(s) in title only field;

**Database:** Embase
**Platform:** Elsevier
**Date Searched:** March 1, 2023
**Date Limits:** 2012–2022
**Other Limits/Filters:** Source: Embase and Embase Classic; A search strategy was used to remove animal studies (i.e., non-human) and specific article types (e.g., conference abstracts, letters, editorials, reviews) that were detailed in our exclusion criteria from the search results).

| **Set** | **Concept** | **Search Strategy** |
| --- | --- | --- |
| #1 | Proteomics | (‘proteomics’/exp OR ‘proteome’/de OR proteom*:ti,ab OR ‘aptamer based’:ti,ab OR ‘antibody based’:ti,ab OR ‘aptamer-based’:ti,ab OR ‘antibody-based’:ti,ab OR somalogic:ti,ab OR Luminex:ti,ab OR Olink:ti,ab OR ‘o link’:ti,ab OR ((biomarker*:ti,ab OR ‘biological marker*’:ti,ab OR ‘biologic marker*’:ti,ab OR ‘biochemical marker*’:ti,ab OR ‘biological marker’/de) AND (protein:ti,ab OR proteins:ti,ab OR ‘Protein’/de)) OR ‘serum marker’:ti,ab OR ‘serum markers’:ti,ab OR ‘serum proteome*’:ti,ab OR ‘serum proteomics’:ti,ab OR ‘serum biomarkers’:ti,ab OR ‘serum biomarker’:ti,ab OR ‘plasma proteome*’:ti,ab OR ‘plasma proteomics’:ti,ab OR ‘plasma marker’:ti,ab OR ‘plasma markers’:ti,ab OR ‘plasma biomarker’:ti,ab OR ‘plasma biomarker*’:ti,ab OR ‘prote-omic*’:ti,ab OR ‘protein omic*’:ti,ab OR proteinomic*:ti,ab) |
| #2 | Heart failure | (‘heart failure’/de OR ‘acute heart failure’/de OR ‘congestive heart failure’/de OR ‘diastolic dysfunction’/de OR ‘experimental heart failure’/de OR ‘forward heart failure’/de OR ‘heart outflow tract obstruction’/de OR ‘heart ventricle failure’/de OR ‘heart ventricle overload’/de OR ‘high output heart failure’/de OR ‘systolic dysfunction’/de OR ‘heart failure*’:ti,ab OR ‘cardiac failure*’:ti,ab OR ‘myocardial failure*’:ti,ab OR ‘systolic failure*’:ti,ab OR ‘diastolic failure*’:ti,ab OR HfrEF:ti,ab OR HfpEF:ti,ab OR HfmrEF:ti,ab OR ‘diastolic dysfunction*’:ti,ab OR ‘systolic dysfunction*’:ti,ab OR cardiomyopath*:ti,ab OR ‘cardiac decompensation*’:ti,ab OR ‘cardiac incompetenc*’:ti,ab OR ‘cardiac insufficienc*’:ti,ab OR ‘cardial decompensation*’:ti,ab OR ‘cardial insufficienc*’:ti,ab OR ‘chronic heart insufficienc*’:ti,ab OR ‘heart decompensation*’:ti,ab OR ‘heart insufficienc*’:ti,ab OR ‘myocardial insufficienc*’:ti,ab OR ‘cardiac backward failure*’:ti,ab OR ‘cardiac stand still’:ti,ab OR ‘decompensatio cordis’:ti,ab OR ‘heart backward failure*’:ti,ab OR ‘heart incompetenc*’:ti,ab OR ‘insufficientia cardis’:ti,ab) |
| #3 |  | #1 AND #2 |
| #4 | Limits: | #3 AND (([embase]/lim OR [embase classic]/lim) AND [2012-2022]/py) |
| #5 |  | #4 NOT (mice:ti,ab OR mouse:ti,ab OR rat:ti,ab OR rats:ti,ab OR dog:ti,ab OR dogs:ti,ab OR pig:ti,ab OR pigs:ti,ab OR piglet:ti,ab OR piglets:ti,ab OR swine:ti,ab OR porcine*:ti,ab OR rodent*:ti,ab OR animal*:ti,ab OR ‘dog’/exp OR ‘pig’/exp OR ‘rodent’/exp OR [animal cell]/lim OR [animal experiment]/lim OR [animal model]/lim OR [animal tissue]/lim OR ape:ti,ab OR apes:ti,ab OR monkey*:ti,ab OR gorilla*:ti,ab OR chimpanzee*:ti,ab OR macaque*:ti,ab OR orangutan*:ti,ab OR pongo:ti,ab OR macaca:ti,ab OR ‘chimpanzee’/exp OR ‘gorilla’/exp OR ‘orangutan’/exp OR ‘Macaca’/exp) |
| #6 |  | #5 NOT ([conference abstract]/lim OR [conference paper]/lim OR [conference review]/lim OR [data papers]/lim OR [editorial]/lim OR [erratum]/lim OR [note]/lim OR [review]/lim OR [short survey]/lim OR [systematic review]/lim OR ‘data paper’/exp OR ‘editorial’/exp OR ‘erratum’/exp OR ‘note’/exp OR ‘short survey’/exp OR ‘review’/exp OR ‘systematic review’/exp OR corrigenda:ti,ab OR corrigendum:ti,ab OR protocol:ti OR protocols:ti OR erratum:ti,ab OR errata:ti,ab OR ‘conference abstract*’:ti,ab OR ‘conference proceeding*’:ti,ab OR symposium*:ti,ab OR editorial:ti,ab OR commentary:ti,ab OR ‘systematic review*’:ti,ab OR ‘integrative review*’:ti,ab) |

**Notes:** Search field tags used in above search.

- /exp = [Emtree](https://www.embase.com/#emtreeSearch/default) indexing terms with explosion to include other terms below it in hierarchy;
- /de = [Emtree](https://www.embase.com/#emtreeSearch/default) indexing terms with explosion turned off to exclude other terms below it in hierarchy;
- :ti,ab = term(s) in title, collection title, abstract, other abstract, or keywords;
- /lim = limited to specific publication type limit;
- [2012-2022]/py = publication year;

**Database:** Web of Science: Core Collection*
**Platform:** Clarivate Analytics
**Date Searched:** March 1, 2023
**Date Limits:** 2012–2022
**Other Limits/Filters:** A search strategy was used to remove animal studies (i.e., non-human) and specific article types (e.g., conference abstracts, letters, editorials, reviews) that were detailed in our exclusion criteria from the search results).

| **Set** | **Concept** | **Search Strategy** |
| --- | --- | --- |
| #1 | Proteomics | TS=((proteom* OR "aptamer based" OR "antibody based" OR "aptamer-based" OR "antibody-based" OR somalogic OR Luminex OR Olink OR "o link" OR ((biomarker* OR "biological marker*" OR "biologic marker*" OR "biochemical marker*") AND (protein OR proteins)) OR "serum marker" OR "serum markers" OR "serum proteome*" OR "serum proteomics" OR "serum biomarkers" OR "serum biomarker" OR "plasma proteome*" OR "plasma proteomics" OR "plasma marker" OR "plasma markers" OR "plasma biomarker" OR "plasma biomarkers" OR "prote-omic*" OR "protein omic*" OR proteinomic*)) |
| #2 | Heart failure | TS=(("heart outflow tract obstruction*" OR "heart ventricle failure*" OR "heart ventricle overload*" OR "heart failure*" OR "cardiac failure*" OR "myocardial failure*" OR "systolic failure*" OR "diastolic failure*" OR HFrEF OR HFpEF OR HFmrEF OR "diastolic dysfunction*" OR "systolic dysfunction*" OR cardiomyopath* OR "cardiac decompensation*" OR "cardiac incompetenc*" OR "cardiac insufficienc*" OR "cardial decompensation*" OR "cardial insufficienc*" OR "chronic heart insufficienc*" OR "heart decompensation*" OR "heart insufficienc*" OR "myocardial insufficienc*" OR "cardiac backward failure*" OR "cardiac stand still" OR "decompensatio cordis" OR "heart backward failure*" OR "heart incompetenc*" OR "insufficientia cardis")) |
| #3 |  | #1 AND #2 |
| #4 | Limits: | #3 AND PY=(2012-2022) |
| #5 |  | #4 NOT TS=(mice OR mouse OR rat OR rats OR dog OR dogs OR pig OR pigs OR piglet OR piglets OR swine OR porcine* OR rodent* OR animal* OR ape OR apes OR monkey* OR gorilla* OR chimpanzee* OR macaque* OR orangutan* OR pongo OR macaca) |
| #6 |  | #5 NOT TI=(editorial OR commentary OR "conference abstract*" OR "conference proceeding*" OR symposium* OR errata OR erratum OR corrigenda OR corrigendum OR protocol OR protocols OR "systematic review*" OR "integrative review*") NOT DT=(Editorial Material OR News Item OR Note OR Book OR Book Chapter OR Excerpt OR Item About an Individual OR Meeting Abstract OR Meeting Summary OR Reprint) |

* Science Citation Index Expanded (SCI-EXPANDED)--1900-present

Social Sciences Citation Index (SSCI)--1900-present

Conference Proceedings Citation Index – Science (CPCI-S)--1990-present

Conference Proceedings Citation Index – Social Science & Humanities (CPCI-SSH)--1990-present

Book Citation Index – Science (BKCI-S)--2005-present

Book Citation Index – Social Sciences & Humanities (BKCI-SSH)--2005-present

Emerging Sources Citation Index (ESCI)--2005-present

Current Chemical Reactions (CCR-EXPANDED)--1985-present

Index Chemicus (IC)--1993-present

**Notes:**

TS = search in Topic fields which includes title, abstract, author keywords, and Keywords Plus fields of a record;

PY = search in Publication Year field of a record;

TI = search in the Title field of a record;

DT = search in the Document Type field of a record;

**Database:** Scopus
**Platform:** Elsevier
**Date Searched:** March 1, 2023
**Date Limits:** 2012–2022
**Other Limits/Filters:** A search strategy was used to remove animal studies (i.e., non-human) and specific article types (e.g., conference abstracts, letters, editorials, reviews) that were detailed in our exclusion criteria from the search results).

| **Set** | **Concept** | **Search Strategy** |
| --- | --- | --- |
| #1 | Proteomics | Title-Abs-Key((proteom* OR {aptamer based} OR {antibody based} OR {aptamer-based} OR {antibody-based} OR somalogic OR Luminex OR Olink OR {o link} OR ((biomarker* OR {biological marker*} OR {biologic marker*} OR {biochemical marker*}) AND (protein OR proteins)) OR {serum marker} OR {serum markers} OR {serum proteome*} OR {serum proteomics} OR {serum biomarkers} OR {serum biomarker} OR {plasma proteome*} OR {plasma proteomics} OR {plasma marker} OR {plasma markers} OR {plasma biomarker} OR {plasma biomarkers} OR {prote-omic*} OR {protein omic*} OR proteinomic*)) |
| #2 | Heart failure | Title-Abs-Key(({heart outflow tract obstruction*} OR {heart ventricle failure*} OR {heart ventricle overload*} OR [72] OR {cardiac failure*} OR {myocardial failure*} OR {systolic failure*} OR {diastolic failure*} OR HFrEF OR HFpEF OR HFmrEF OR {diastolic dysfunction*} OR {systolic dysfunction*} OR cardiomyopath* OR {cardiac decompensation*} OR {cardiac incompetenc*} OR {cardiac insufficienc*} OR {cardial decompensation*} OR {cardial insufficienc*} OR {chronic heart insufficienc*} OR {heart decompensation*} OR {heart insufficienc*} OR {myocardial insufficienc*} OR {cardiac backward failure*} OR {cardiac stand still} OR {decompensatio cordis} OR {heart backward failure*} OR {heart incompetenc*} OR {insufficientia cardis})) |
| #3 |  | #1 AND #2 |
| #4 | Limits: | #3 AND (LIMIT-TO(PUBYEAR, 2022) OR LIMIT-TO(PUBYEAR, 2021) OR LIMIT-TO(PUBYEAR, 2020) OR LIMIT-TO(PUBYEAR, 2019) OR LIMIT-TO(PUBYEAR, 2018) OR LIMIT-TO(PUBYEAR, 2017) OR LIMIT-TO(PUBYEAR, 2016) OR LIMIT-TO(PUBYEAR, 2015) OR LIMIT-TO(PUBYEAR, 2014) OR LIMIT-TO(PUBYEAR, 2013) OR LIMIT-TO(PUBYEAR, 2012)) |
| #5 |  | #4 AND NOT Title-Abs-Key(mice OR mouse OR murine* OR rat OR rats OR rodent* OR dog OR dogs OR pig OR pigs OR piglet* OR swine OR porcine* OR animal* OR ape OR apes OR monkey* OR gorilla* OR chimpanzee* OR macaque* OR orangutan* OR pongo OR macaca) AND NOT INDEX("Pan paniscus" OR "Pongo" OR "Macaca" OR "Gorilla gorilla" OR Dogs OR Swine OR Rodentia OR "Models, Animal" OR "Animal Experimentation") |
| #6 |  | #5 AND NOT DOCTYPE(ab OR bk OR ch OR cp OR cr OR bz OR dp OR ed OR er OR mm OR no OR pr OR rp OR re OR sh) AND NOT Title(editorial OR commentary OR {conference abstract*} OR {conference proceeding*} OR symposium* OR errata OR erratum OR corrigenda OR corrigendum OR protocol OR protocols OR {systematic review*} OR {integrative review*}) |

Grey Literature Searches

**Clinical Trials.gov**

**Resource:** clinicaltrials.gov

**Platform:** US National Library of Medicine

**Date Searched:** May 1, 2023

**Limits Used:** None; Use Advanced Search

Other Terms: (Proteomic* OR somalogic OR Luminex OR Olink OR "o link" OR aptamer OR (protein* AND biomarker*))

AND

Condition: (“heart failure” OR “systolic failure” OR “diastolic failure” OR HFrEF OR HFpEF OR HFmrEF)

**Cochrane Library: CENTRAL (Wiley & Sons)**

**Resource:** Cochrane Library: CENTRAL

**Platform:** Wiley & Sons

**Date Searched:** May 1, 2023

**Limits Used:** Publication Year: 2012–2022; Content Type: Trials

#1 ([mh "Proteomics"] OR [mh "Proteome"])

#2 (proteom* OR "aptamer based" OR "antibody based" OR "aptamer-based" OR "antibody-based" OR somalogic OR Luminex OR Olink OR "o link"):ti,ab,kw

#3 (biomarker* AND protein*):ti,ab,kw

#4 #1 OR #2 OR #3

#5 ([mh ^"Heart Failure"] OR [mh "Heart Failure, Diastolic"] OR [mh "Heart Failure, Systolic"])

#6 ("heart failure" OR "systolic failure" OR "diastolic failure" OR HFrEF OR HFpEF OR HFmrEF):ti,ab,kw

#7 #5 OR #6

#8 #4 AND #7

#9 #8 AND Cochrane Library publication date from Jan 2012 to Dec 2022, in Trials (Word variations have been searched)
