# Supplementary material for: Proteomics for heart failure risk stratification: a systematic review
Source: BMC Med. 2024 Jan 25;22:34. doi: 10.1186/s12916-024-03249-7 (PMC10809595; doi:10.1186/s12916-024-03249-7)
Supplement: Supplementary file 2 — Additional file 2. Risk of bias assessment using the ROBINS-E tool. [file 12916_2024_3249_MOESM2_ESM.docx]

**Proteomics for Heart Failure Risk Stratification: A Systematic Review**

**Additional File 2.**

**Fig. S1. Risk of bias assessment using the ROBINS-E tool.**


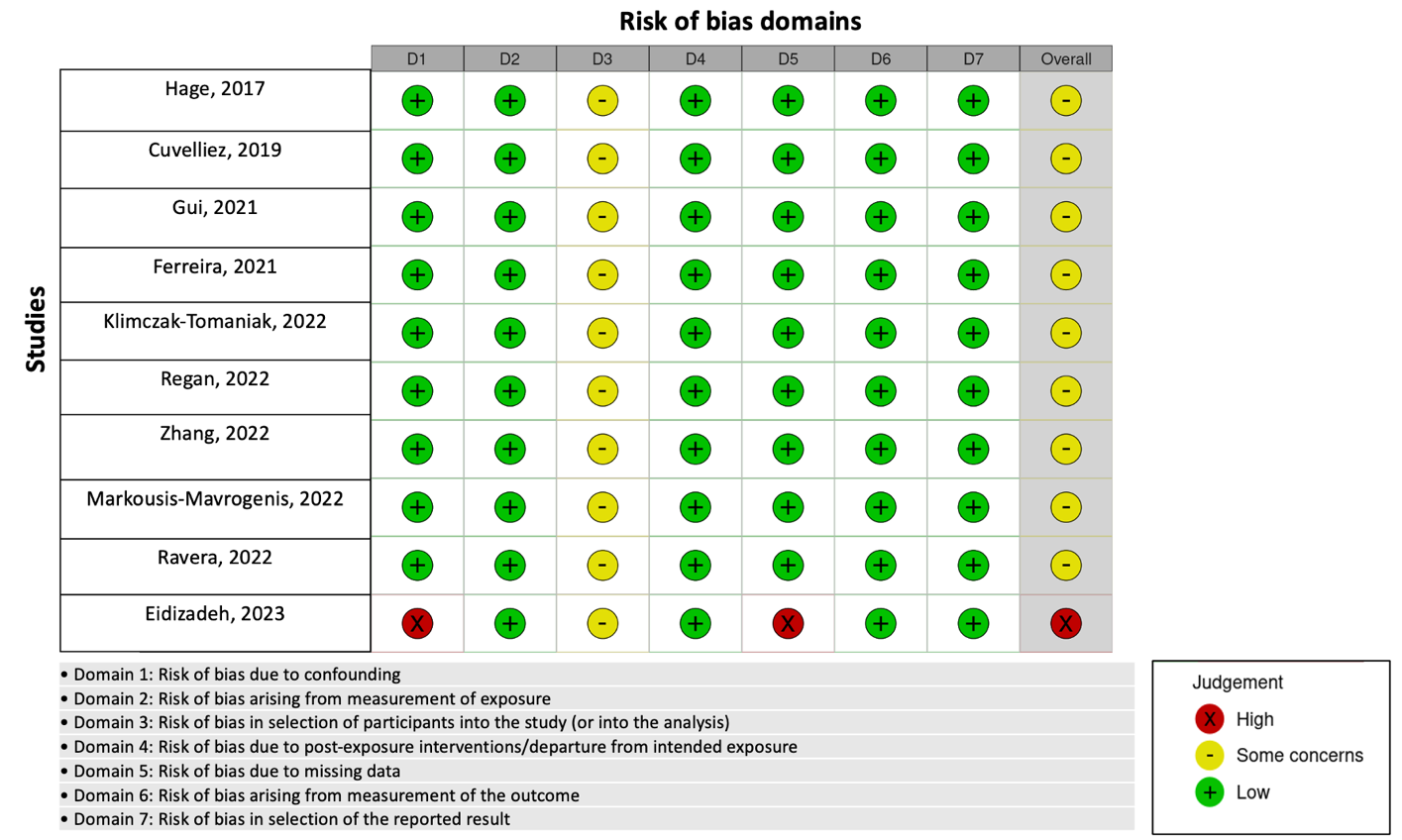


Overall risk determination: (i) High risk is defined by at least one domain with a high risk of bias, with no domains classified as Very high risk OR when several domains have some concerns, leading to an additive judgment of High risk of bias; (ii) Some concerns (Moderate risk) is defined by at least one domain having some concerns, and no domain classified as High risk or Very high risk; (iii) Low risk is defined by a low risk of bias in all seven domains except for concerns about residual confounding in Domain 1 (confounding) and low risk of bias in all other domains.
